# The Emotional Anatomy of Diagnosis: A Medical Humanities Approach to Empathy in Pathology

**DOI:** 10.3390/diagnostics15151842

**Published:** 2025-07-22

**Authors:** Iuliu Gabriel Cocuz, Raluca Niculescu, Maria Cătălina Popelea, Adrian-Horațiu Sabău, Maria-Elena Cocuz, Martin Manole, Alexandru-Constantin Ioniță, Giordano Altarozzi, Maria Tătar-Dan, Ovidiu Simion Cotoi, Dorina Maria Pașca

**Affiliations:** 1 Pathophysiology Department, “George Emil Palade” University of Medicine, Pharmacy, Sciences and Technology of Targu Mures, 540142 Targu Mures, Romania; iuliu.cocuz@umfst.ro (I.G.C.); ovidiu.cotoi@umfst.ro (O.S.C.); 2 Pathology Department, Mures Clinical County Hospital, 540011 Targu Mures, Romania; popelea.maria@gmail.com; 3 Histology Department, “George Emil Palade” University of Medicine, Pharmacy, Sciences and Technology of Targu Mures, 540142 Targu Mures, Romania; 4 Fundamental Prophylactic and Clinical Disciplines Department, Faculty of Medicine, Transilvania University of Brasov, 500003 Brașov, Romania; maria.cocuz@unitbv.ro; 5 Clinical Pneumology and Infectious Diseases Hospital of Brasov, 500174 Brasov, Romania; 6 Faculty of Medicine, “George Emil Palade” University of Medicine, Pharmacy, Sciences and Technology of Targu Mures, 540142 Targu Mures, Romania; martinmanolemd98@gmail.com (M.M.); alexionita2@gmail.com (A.-C.I.); 7 Science and Letters 1 Department, Petru Maior Faculty of Sciences and Letters, “George Emil Palade” University of Medicine, Pharmacy, Sciences and Technology of Targu Mures, 540142 Targu Mures, Romania; giordano.altarozzi@umfst.ro (G.A.); maria.tatar-dan@umfst.ro (M.T.-D.); 8 Ethics Department, “George Emil Palade” University of Medicine, Pharmacy, Sciences and Technology of Targu Mures, 540142 Targu Mures, Romania

**Keywords:** pathology, empathy, medical personnel, emotional well-being, medical humanities

## Abstract

**Background/Objectives:** Pathology is often perceived as a technical medical specialty that lacks direct contact with the patient. However, oncological histopathological diagnosis requires a high degree of moral and emotional responsibility. The objective of this study was to investigate how empathy is manifested toward the “invisible” patient, the emotional impact on pathology staff, and potential repercussions in their personal lives. **Method:** We conducted a descriptive, cross-sectional study with a quantitative component, using an anonymous 22-item questionnaire among Romanian pathologists and medical personnel working in pathology services. The questionnaire was focused on three research directions: professional empathy in the absence of direct patient contact, the emotional impact of oncologic diagnosis on medical personnel in pathology departments, and the carryover of emotions from professional to personal life. A total of 165 respondents were included in the study (physicians, technicians, registrars). **Results:** Most of the respondents consider that the absence of the patient’s direct contact does not cancel the empathy, this being felt in a cognitive and more natural way. Over 60% of the respondents see oncologic histopathological diagnosis as an emphatic medical act. Over 80% of the respondents experience a sense of emotional responsibility and 70% consider that professional training does not include adequate emotional support. There is a high interest in empathy and psychological support. The professional activity of a pathologist may influence sleep, dreams, and the perception on their own health status. Diagnosing pediatric or young patients is perceived as particularly emotionally challenging. Collegial support is moderate and discussion about professional stress is rare. **Conclusions:** Empathy is present and relevant in pathology, despite the absence of direct patient interaction. Oncological diagnostics has a significant emotional impact on pathology department personnel, with the need to acknowledge the emotional dimension of the profession and to integrate psychological support mechanisms into pathology practice.

## 1. Introduction

Medicine is more than a science; it is a practice full of humanity, where suffering, communication, and empathy must relate in any specialty, even in pathology, a specialty often perceived as distant and cold towards the patient [1]. In this profession, deep contact with the patient is absent, and interaction with the patient is based on contact with the patient’s tissues and clinical data [2]. Pathology is directly linked to the trajectory of human suffering through the high responsibility that the pathologist has over the oncological histopathological diagnosis, a decision that can profoundly influence the course of a life. Studies show that the emotional impact of these decisions is sometimes even stronger than in specialties where direct contact with the patient is made, causing empathic exhaustion and moral distress [3].

Pathologists may develop burnout or professional isolation in the absence of real psychological support mechanisms. At the same time, empathy can also be affected by the formative process a physician goes through [4,5].

Medical humanities is an essential component of modern medical education, where, in addition to theoretical and practical knowledge, critical reflection and human understanding are also sought. At the same time, medical humanities also brings to light the imbalances that advanced digitalization and technologization of medicine create, thus offering a humanistic side to algorithms and the doctor–patient relationship. [6].

The present study represents a premiere in Romania and in the specialized literature because at this moment, this is probably the only study conducted on the empathy that medical personnel in pathology services feel towards oncological diagnosis, as well as the repercussions of oncological diagnosis on medical personnel.

The objective of this study was to inspect, analyze, and understand the empathic and emotional dimension of the staff working in pathology services in the context of diagnosing oncological diseases, in the absence of direct contact with the patient. This study aims to investigate how empathy is manifested towards the “invisible” patient, the emotional impact felt in everyday medical activity, the possible transposition of these feelings and the impact in personal life, and the adaptation strategies used by medical staff in pathology services.

This study was designed to respond to an essential component of the medical profession, namely, empathy towards the patient and towards the patient’s life. In pathology, the patient being often unseen, empathy exists, and the mental health of the staff must be in direct harmony with the diagnosis.

## 2. Materials and Methods

This study was a descriptive, cross-sectional study with a quantitative component, based on a research instrument in the form of a self-administered questionnaire sent to the participants. It aimed to explore the perceptions, feelings, and emotional impact associated with oncological diagnostic activity within pathology services.

The questionnaire was addressed to active staff within pathology departments composed of resident physicians, specialists, and senior physicians within the pathology medical specialty, laboratory assistants, laboratory technicians, medical examiners, caregivers, and medical registrars.

The questionnaire was distributed through the Romanian organization of pathologists, (Uniunea Patologilor din România—UNIPAT), through groups on electronic messaging services (733 members), respectively, by email, at the initiative of the president of the association.

The inclusion criteria for participants in this study were current activity in a pathology department in Romania or outside Romania, but the respondent is Romanian; informed (implicit) consent of the respondent for the processing of data obtained by completing the questionnaire; and classification in one of the mentioned professional categories.

The questionnaire was anonymous, structured on 22 research items, 3 of which were represented by socio-professional identification elements (age, experience in the field measured by the number of years of professional activity, and professional category). The other 19 items (Table 1) were represented by 3 research directions: professional empathy in the absence of direct patient contact, the emotional impact of oncologic diagnosis on medical personnel in pathology departments, and the carryover of emotions from professional to personal life. The stratification of the questions by research areas is presented in Table 1.

The questions were formulated in a clear manner, using closed wording, with 4 answer options, to allow both statistical analysis and maintaining a high degree of objectivity. The items were written in accessible, rigorous language, avoiding vague terms.

The response collection period was 4 weeks (1–31 May 2025). Participation was voluntary and anonymous, and completing the questionnaire represented the implicit acceptance of each participant for the completion and processing of the data obtained. There were 165 unique responses collected. The questions were answered by all the participants except for 3 questions: Question 7 (164 answers), Question 11 (164 answers), and Question 16 (164 answers). The response rate was 22.51% (165 answers).

This study complies with all ethical and confidentiality principles. No personally identifiable data (name, email address, workplace) was collected. The completion of the questionnaire was preceded by clear information regarding the purpose of the research, the title of the paper, the identity of the author, and the use of the data exclusively for academic purposes.

Data analysis was performed by analyzing the data obtained in tabular format. For numerical analysis and descriptive statistics of the data, the Microsoft Excel platform from the Office suite was used. The results obtained were represented in tabular or graphical form.

For the statistical analysis of the data, GraphPad Prism 10.0 (GraphPad Software, San Diego, CA) was used. From the point of view of statistical tests, the Chi-square test was used to analyze the association between categorical variables in the questionnaire. Statistical analyses were performed with a 95% confidence level. The result was considered statistically significant if the *p*-value was less than 0.05 (*p* < 0.05).

Limitations of this study: Given the study design, the study data reflects the perceptions and experiences of the participants at a single point in time. The participants completed the questionnaire voluntarily, which may lead to auto-selection bias. This may be due to differences in motivation to participate. At the same time, there is a subjective perception of the questions, depending on personal experience or other variables, which may affect the consistency of the responses.

## 3. Results

Table 2 presents the socio-demographic characteristics of the respondents, distributed by age group, medical staff category, and years of professional experience. Table 3, Table 4, Table 5 and Table 6 present the answers provided by the respondents in percentage and numerical data.

**Figure 1:**
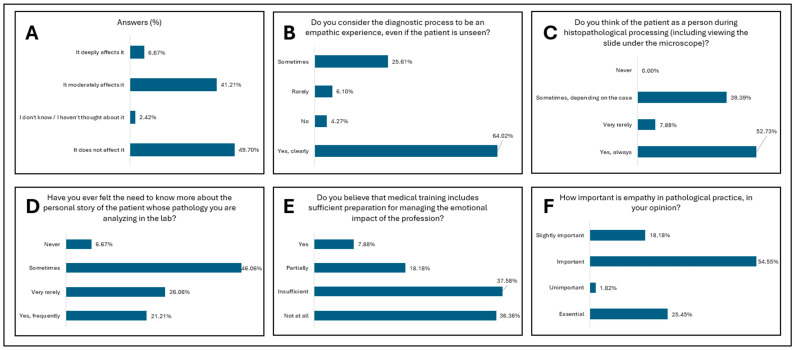
Research direction: Professional empathy in the absence of direct patient contact. (**A**). Perception of the impact of the absence of direct patient contact on the level of professional empathy among respondents. (**B**). Perception of the empathic nature of the diagnostic act in the absence of direct patient interaction among respondents. (**C**). The professional’s relationship with the patient during the histopathological process among respondents. (**D**). The need to understand the patient’s biographical dimension in histopathological analysis among respondents. (**E**). Perception of the degree of professional training in managing the emotional impact of medical practice among respondents. (**F**). The importance attributed to empathy in the professional practice of pathologists among respondents.

**Figure 2:**
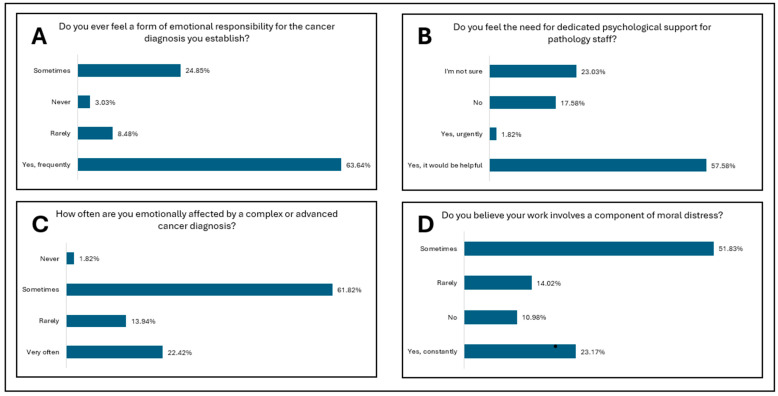
Research direction: The emotional impact of oncologic diagnosis on medical personnel in pathology departments. (**A**). Experience of emotional responsibility associated with establishing an oncologic diagnosis among respondents. (**B**). Perceived need for specialized psychological support among pathology department staff according to respondents. (**C**). Frequency of emotional impact in cases of complex or advanced oncologic diagnoses among respondents. (**D**). Perception of the existence of moral distress in professional practice among respondents.

**Figure 3:**
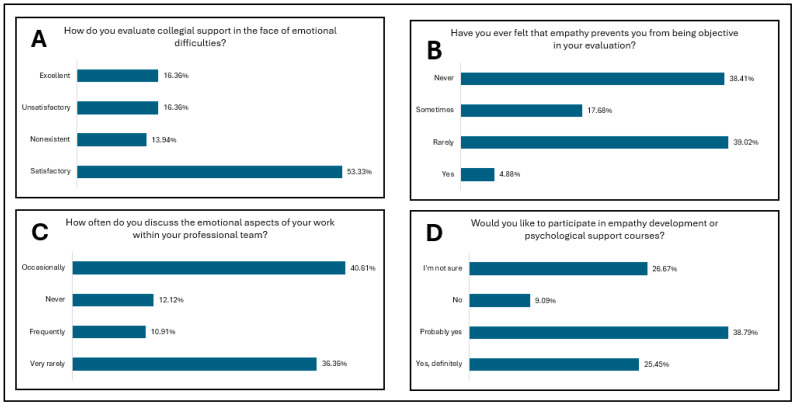
Research direction: The emotional impact of oncologic diagnosis on medical personnel in pathology departments. (**A**). Evaluation of collegial support in the context of emotional challenges of the profession among respondents. (**B**). Perception of empathy’s influence on diagnostic objectivity among respondents. (**C**). Frequency and openness of discussions regarding the emotional impact of professional activity within the team according to respondents. (**D**). Interest in participating in training programs on empathy development and psychological support among respondents.

**Figure 4:**
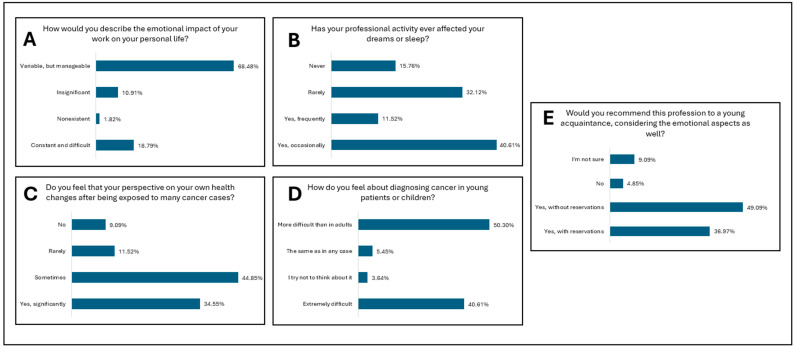
Research Direction: The carryover of emotions from professional to personal life. (**A**). Assessment of the emotional impact of professional activity on personal life among respondents. (**B**). Influence of professional activity on sleep and dream content among respondents. (**C**). Changes in self-perception of personal health following frequent exposure to oncologic cases among respondents. (**D**). Emotional reactions to establishing a cancer diagnosis in pediatric or young patients among respondents. (**E**). Willingness to recommend the profession of pathologist in light of awareness of its emotional burden among respondents.

Figure 1 presents the study results for the first research direction—professional empathy in the absence of direct patient contact. Figure 2 and Figure 3 present the study results for the second research direction—the emotional impact of oncologic diagnosis on medical personnel in pathology departments. Figure 4 presents the study results for the third research direction—the carryover of emotions from professional to personal life.

Table 7 presents the correlation of statistically significant coefficients between socio-demographic characteristics of the participants and responses to the key survey questions.

## 4. Discussion

Pathology is a medical branch that interacts empathetically with any pathology of the non-present patient (no direct patient contact), that looks at each patient at a structural level, and that sees beyond the patient, inside him, both literally and figuratively. A person’s empathy is innate and adapted through their own experiences throughout life [4]. Empathy is a basic professional skill in patient-centered medicine, where the patient’s suffering is understood through its cellular structure and clinical context [7]. The definition of empathy remains complex and is discussed in specialized literature and is still conceptually ambiguous in the current literature. Empathy involves two components, one of understanding the personal experiences of each patient and another emotional, of feeling affection for him [3]. Specialized literature suggests that empathy can be viewed as a stable trait, modulated by childhood experiences, but also a trait that can fluctuate depending on the professional context, stress level, and social interactions [4,8].

This study addresses the branch of pathology regarding oncological diagnosis, a diagnosis that can significantly impact patients and can influence decisions, experiences, or feelings. Thus, this study aimed to analyze the empathy of the doctor beyond the microscope, in the process of making the diagnosis.

The socio-demographic analysis of the respondents shows the dynamics of the responses depending on age, medical staff category, and experience in this specialty. Other studies have shown that other factors, such as being female and the presence of children, increase empathy scores. This suggests that life experiences contribute to the formation of a much deeper empathetic relationship [9]. Table 2 shows, from the age point of view, that most of the respondents were part of the age category 31–40 years, followed by 51–60 years and 41–50 years. From the point of view of the personnel category, most respondents were primary care physicians, followed by specialists and residents. Regarding other categories, the questionnaire was also completed by medical registrars and laboratory assistants. The most common range of specialty experience was in the 11–20 years of experience category, followed by the 5–10 years and under 5 years categories.

The first research direction addressed in this study was professional empathy in the absence of the patient and is composed of 6 research items. The perception of the impact of the absence of direct contact with the patient for the stratification of the level of professional empathy (Figure 1A and Table 3) indicates that almost half of the respondents believe that the absence of the patient does not influence professional empathy, even if the patient is not seen, although a significant part of the respondents believe that empathy is moderately influenced. This may reflect a common professional trait in pathology, where the relationship with the patient is mediated much more strongly by clinical data and microscopic images. In this context, empathy could have a more cognitive or technical expression, less dependent on direct interaction. This perception corresponds to the cognitive dimension of empathy, one of the four dimensions of empathy described by Morse—emotional, moral, cognitive, and behavioral—which act in direct accordance with medical practice [10]. The predominantly cognitive approach must be balanced with authentic personal reflection and moral involvement to manage the lack of practical application of empathy [1]. Statistical analysis indicates significant associations between the perception of this influence and professional degree (*p* = 0.0016). This suggests that professional experience influences the understanding and integration of empathy in the establishment of the oncological histopathological diagnosis (Table 6). Given that for any patient, the experience of the medical act and diagnosis must reflect the doctor’s empathy towards the patient’s suffering, over 60% of respondents consider that even when the patient is not seen, the diagnostic experience is an empathetic one where medical dynamism, humanistic values, and science meet to establish a diagnosis of certainty. In comparison, a very small percentage of respondents consider that the oncological histopathological diagnosis process is not an empathetic experience (Figure 1B and Table 3). Although the patient is physically absent, the empathetic relationship is mediated through a subtle mechanism of emotional reflection. The doctor’s disposition influences how the patient’s experience is interpreted. [11] This opinion is significantly differentiated between resident physicians and specialist/primary physicians (*p* = 0.0446), suggesting an evolution in the empathy reporting mode with experience and professional maturation (Table 6).

Figure 1C and Table 3 demonstrate that over half of the respondents always visualize the patient as a person and as a human being within the oncological histopathological diagnosis, even if he is not seen and is not physically present. Almost 40% of them consider that, depending on the case, they react with humanity to the histopathological diagnosis. A statistically significant result (*p* < 0.0001 for resident versus specialist or consultant, *p* = 0.000065 for personal category, and *p* = 0.0267 for personal category) was obtained in relation to the question regarding thinking about the patient as a person during histopathological processing, highlighting a high degree of internalization of the human perspective in the diagnostic process in accordance with the professional category and professional maturity (Table 6). The biographical dimension is felt by the staff within the pathology services by the fact that there is a frequent curiosity about the person behind the histological slide. The emphasis falls on the desire to know more information, to assimilate the patient as a whole, and to diagnose the disease in relation to the existential dimension of the patient (Figure 1D and Table 3). A statistically significant result (*p* = 0.0151) was obtained regarding the resident versus specialist or consult regarding the need to know more about the story of the patient. This signifies that the more advanced in his carer the pathologist is, the more curious about the human dimension he is (Table 6). Reflecting, empathy relates to the cognitive dimension and manifests itself through communicative models, being placed in the three phases of empathy: resonance, expression, and reception. All these characteristics are essential in building a connection with the patient, even unseen [10]. The emotional impact of a medical diagnosis, especially an oncological diagnosis, on the doctor exists; is felt permanently and should not be neglected. In this context, the concept of compassion can be developed. Fatigue is another form of burnout that occurs after repeated exposure to patients’ suffering, even when the patient is not seen [12]. Specialized medical training during the years of undergraduate studies for medical personnel (between 4 and 6 years) and medical residency training (for doctors) represents over 10 years of continuous medical training and education for the medical profession. Even though the permanent study of medical science is dominant in this profession, there are complementary disciplines, among which is doctor–patient communication, which has the role of developing the empathy that the doctor transmits to the patient. However, in our study, over 70% of the respondents consider that the emotional impact of an oncological diagnosis is not based on solid training within professional training. This can be managed by introducing practical subjects that develop the ability of a future doctor to know what empathy is, how it can be managed and applied, and the impact of the oncological diagnosis to be adequately managed for both the diagnosing physician and the patient (Figure 1E and Table 3). The significant differences between physicians and other categories of personnel (*p* = 0.000007), professional experience (*p* = 0.0118), and resident versus specialist or consultant (*p* = 0.0021) emphasize the need for curricular interventions in the training of future young health professionals (Table 6). Residents and younger specialists more frequently report a lack of emotional training during professional training. This trend is also reported in other studies, which demonstrate a tendency for empathy to fade during residency training due to the extremely busy and stressful period. [4,13]. This suggests the likelihood of vulnerability in the early years of a career but also the need for formalized support. The difference is made by the accounts of more experienced staff who do not feel this lack so strongly. There is a high awareness among the younger generations of the importance of emotional preparation (Table 6).

The research direction concludes its journey by focusing on the importance of empathy in the professional practice of staff in pathology services. Thus, over three-quarters of the respondents considered that empathy in professional practice is important and essential for establishing a diagnosis of certainty and correctness and starts the path towards increasing the patient’s quality of life (Figure 1F and Table 3). This vision is in direct agreement with the narrative medicine model, which describes the fact that the efficiency of the medical act is directly related to the doctor’s ability to interpret each case with empathy, professionalism, and reflection [14].

The second research direction investigates the emotional impact of oncological diagnosis on medical staff in pathology services. The direction is structured on eight research items that strengthen the emotional perspective on a diagnosis for an unseen patient. Medical empathy in the face of an oncological diagnosis is not only an element of communication but can directly influence the quality of the medical act. In addition to reducing patient anxiety, empathy also improves the professional satisfaction of doctors [3,15]. Figure 2A and Table 4 reflect the experience of emotional responsibility associated with establishing an oncological diagnosis. Unlike medical staff who are subject to burnout, models based on artificial intelligence demonstrate a constant line for empathy through the absence of fatigue and emotional affect [14]. Thus, among the respondents, emotional responsibility is felt by almost all the respondents, the vast majority considering that they frequently feel this responsibility, followed by those who consider that this emotional responsibility exists but is felt infrequently, often rarely or sometimes. The statistical results confirm significant differences depending on professional category (*p* = 0.0000049), indicating a clear differentiation between doctors and other professional categories (Table 6). Emotional responsibility manifests itself differently for each member of the pathology services community, from the technician or assistant who processes the tissue sample to the doctor who makes the diagnosis. Regarding pathologists, they establish an extremely important diagnosis without having direct communication with the patient. For this reason, an imbalance between responsibility and control may occur. This imbalance is described in the literature as an important factor in the emergence of moral distress. Personnel from various medical categories interpret emotional responsibility for establishing an oncological diagnosis differently, with differences between specialists and primary care physicians compared to laboratory personnel who feel this aspect less often. Figure 2B and Table 4 show the perceived need for specialized psychological support among pathology laboratory staff. Specialized psychological support, a branch that is not amplified to its maximum levels in Romania, would reduce the degree of emotional stress over an oncological diagnosis, multiplied hundreds of times by the multitude of oncological diagnoses that the pathologist establishes. Working in an organizational climate where empathy is positively valued and where positive professional models are present, empathy can be protected, and thus a patient-centered professional identity can be developed, even if the patient is unseen [4]. Over half of the respondents believe that such specialized psychological support would be useful for them, but it does not exist; almost a quarter stated that they do not know if this would be useful. In addition to psychological support, the tendency not to prioritize personal care is an aggravating factor. Staff who pay little attention to their own emotional health may be at increased risk of burnout and impaired empathy. [16,17]. It can be observed from Table 6 that specialist or primary care physicians more frequently see each case as a person, compared to residents or laboratory personnel. This correlation demonstrates the high level of clinical experience in diagnostic decisions. The differences noted between professional experience and the perception of the case as a person show that experienced personnel (more than 20 years of experience) always think of the case as a person compared to respondents with less experience. From this, one can observe a professional maturation developed with experience but also a deeper integration of the human dimension. The internalization of moral responsibility can be seen and felt through the prism of the implications of the oncological diagnosis on the patient’s life.

In addition to the emotional responsibility felt by the staff in the pathology services, another human side is the emotional effect and the transformation of responsibility into psychological stress. Various environmental factors, such as a high workload, bureaucracy, or lack of support from the organization where the staff works, can contribute to the decrease in empathy and the emergence of the burnout phenomenon [4,18]. Figure 2C and Table 4 show us that over 80% of the respondents consider that they are emotionally affected by the complex or advanced oncological diagnoses they establish, either very often or sometimes, but that this effect exists and is present. Through developed empathy, a deep understanding of the medical context can be achieved, as well as the development of a relationship with therapeutic implications, even indirect, between the pathologist and the patient [19]. The loss of an empathic connection with the patient, even if the patient is unseen, can lead to professional exhaustion. Both patients and doctors suffer when empathy is absent from the medical act [3,20]. Moral distress is a negative emotional response when doctors know the morally correct action but do not implement it due to internal or external constraints. This leads to phenomena such as burnout, low job satisfaction, and nervousness. Figure 2D and Table 4 demonstrate the perception of moral distress in the professional practice of the respondents. Over half of the respondents report that they sometimes felt a certain degree of moral distress in their personal practice, and almost a quarter of the respondents report that they feel a constant degree of moral distress. This is comparable with the other items related to the responsibility and emotional distress of the staff. In terms of emotional distress, a significant influence can be observed depending on the professional category. Moral distress occurs especially in situations where empathy is confused with sympathy, where sympathy implies an affective side, while empathy shows understanding, but with the maintenance of objectivity [8]. Empathy does not refer to the absorption of the emotions and feelings of the patient but to understanding the patient’s suffering without integrating it into a personal experience. This distinction is necessary to maintain the inner balance of the medical staff [7,21].

The community in which pathology staff work is particularly important, and collegiality and teamwork are essential elements for a quality final diagnosis. Figure 3A and Table 5 assess peer support in the context of emotional challenges among the respondents. Thus, over half of the respondents considered peer support for emotional challenges to be satisfactory. At the same time, there are relatively equal margins between excellent, unsatisfactory, and non-existent support. Peer support can be a support for compassionate prevention fatigue because it can provide a framework for emotional validation and reduce professional isolation [12]. Figure 3B and Table 5 analyze how objectivity in the histopathological diagnostic process can be affected by empathy. Most respondents believe that empathy does not affect objectivity or if it does, it happens in quite rare cases. It is worth noting that a fifth of the respondents believe that empathy can affect objectivity always or at least sometimes. An important thing to note is that there must be a series of healthy boundaries of empathy, otherwise maladaptive empathy may appear, with the possibility of affecting the objectivity of the medical act and emotional exhaustion [5,22,23].

In addition to peer support on the emotional burden and the relationship between objectivity and empathy in the development of an oncological diagnosis, Figure 3C and Table 5 also analyze how the emotional aspects of the profession are discussed within the professional team, including outside the laboratory in which the respondent works. Almost half of the respondents discussed aspects related to the emotional impact of professional activity, but almost as many said that they do not discuss these aspects or discuss them very rarely. A small number of respondents reported that they discuss these aspects within the professional team frequently, but the dominant fact remains that experiences and emotions from the same professional category are rarely shared with colleagues in the same field. The research direction is concluded with the analysis of the intention of staff in pathology laboratories to participate in a training program on the development of empathy and psychological support. The results are questionable, given that over half of the respondents considered yes, or probably yes; on the other hand, slightly less than half do not know or do not want to participate in such programs. Thus, we can say that depending on age, professional status, and experience in pathology, there is indeed a difference in what is defined as empathy, emotional load, and psychological support (Figure 3D and Table 5).

The last research direction focused on the transposition of emotions from professional life into personal life among the respondents. The focus area is composed of five research items, focused on the combination of professional and personal, related to the fact that everyday staff in pathology services encounter serious and life-threatening pathologies. Figure 4A and Table 6 report on the impact of professional activity on personal life. Although a small part of the respondents considered it constant and difficult, most respondents considered the impact to be variable but manageable, and very few of them considered it to be either non-existent or insignificant. Most of the emotions experienced in the conscious are transposed into the subconscious and are felt especially when the brain enters a state of wakefulness through sleep, followed by the period of REM sleep (rapid eye movement sleep), that is, during the dream period, many of these emotions are transposed. Figure 4B and Table 6 explore how professional activity affects sleep and dreams, where the results show that most respondents believe that rest, sleep, and dreams are affected either frequently or occasionally by professional activity. On the other hand, a smaller part of the respondents believed that their sleep and dreams are affected rarely or never. The specialized literature shows that fatigue and chronic stress can negatively influence the ability to respond with empathy, which can lead to influencing the quality of the medical act and the well-being of the staff [4].

Personal health is of the utmost importance for professional activity to be carried out in a balanced and productive manner. In the case of staff in pathology services, there is a situation in which the perception of one’s own health is influenced by frequent exposure to oncological cases. Figure 4C and Table 6 analyze the perception that the respondents had regarding their own health. Thus, most respondents considered that their perception is sometimes influenced, and, similarly, many consider that perception is significantly influenced following oncological diagnoses developed in professional activity. Compared to other research items, for this item, over 80% of the respondents considered that the perception of one’s own health can be directly influenced.

The moment of diagnosing a pediatric patient with an oncological condition, whether from the field of hematological diseases or from the quarter of solid tumors, involves a significant emotional burden and a high level of responsibility for the diagnosis. Unfortunately, as in any medical specialty, pediatric pathology is vast and numerous, including in the oncological field. Thus, Figure 4D and Table 6 report the emotional reactions of the staff in the pathology services in the case of the diagnosis provided to a young or pediatric patient. Half of the respondents considered that diagnosis is more difficult in adults, and over 40% considered that diagnosis in pediatric patients is extremely difficult. An emotional defense barrier, existing in the case of a small number of the respondents, is represented by the attempt not to think about the patient at the time of diagnosis.

The last item analyzed in the research direction is the possibility of recommending the specialty of pathology, in terms of the emotional load that doctors and staff in pathology services feel, to a young person they know (Figure 4E and Table 6). In terms of a response of either strongly or tentatively recommend, over 80% of the respondents would recommend the specialty to a future doctor, emphasizing the dynamics of this specialization. However, there are recent studies that suggest that in the absence of a balance between professional satisfaction and emotional involvement, only a small part of medical staff feels a state of professional fulfillment [16,22,24].

## 5. Conclusions

Empathy in pathology goes beyond the absence of physical contact with the patient, having a cognitive and professional manifestation side, which emphasizes moral responsibility and the depth of the diagnostic act in the oncological sphere. The unseen patient does not equate with the absence of empathy in the medical act but transforms it into a true and profound form of identification at a human and intellectual level, in direct relation to the patient’s suffering transmitted through tissue samples. The oncological histopathological diagnosis creates an intense emotional responsibility among the staff in pathology services, regardless of the degree of experience in the field and, thereby, the imperative need to recognize the emotional component of the profession. Emotional preparation within professional training is deficient, and this lack is perceived especially at the level of resident doctors, emphasizing the need to introduce disciplines regarding empathy in the medical act within professional training. Mature empathy is especially present among experienced personnel and has a deep integration into the professional act, signifying the internalization of the human dimension of illness and suffering with career advancement. Psychological support provided to pathology staff is lacking, although it is perceived as necessary by over half of the respondents. It often remains a non-existent or inadequately accessed resource in the Romanian medical system. Advanced or complex oncological histopathological diagnosis has a strong emotional impact on the staff of pathology services, an impact that transcends into personal life by affecting sleep, dreams, and the perception of one’s own health. Talking about and discussing professional stress and emotions experienced in the professional field is still considered a sensitive topic in professional groups. This creates an ideology of harmful psychological isolation related to the suffering experienced individually. The oncological histopathological diagnosis among pediatric or young patients produces an increased emotional burden, distinct and often difficult to manage compared to the adult patient, thus leading to the need for a differentiated psychological approach compared to the adult patient. Pathology is a specialty where emotional challenges and stress are inherent, but most staff would recommend the pathology specialty, where, in addition to all the emotions, there is a professional vocation supported by the deep meaning of the work of professionals in this specialty.

## Figures and Tables

**Table 1:** Structure of the questionnaire questions by research directions.

No. Crt.	Order in the Questionnaire	Question Statement
Research direction: professional empathy in the absence of direct patient contact		
1	4	To what extent do you believe that the lack of direct contact with the patient affects your professional empathy?
2	7	Do you consider the diagnostic process to be an empathic experience, even if the patient is unseen?
3	8	Do you think of the patient as a person during histopathological processing (including viewing the slide under the microscope)?
4	14	Have you ever felt the need to know more about the personal story of the patient whose pathology you are analyzing in the lab?
5	18	Do you believe that medical training includes sufficient preparation for managing the emotional impact of the profession?
6	20	How important is empathy in pathology practice, in your opinion?
Research direction: the emotional impact of oncologic diagnosis on medical personnel in pathology departments		
7	5	Do you ever feel a form of emotional responsibility for the cancer diagnosis you establish?
8	9	Do you feel the need for dedicated psychological support for pathology staff?
9	10	How often are you emotionally affected by a complex or advanced cancer diagnosis?
10	11	Do you believe your work involves a component of moral distress?
11	15	How do you evaluate collegial support in the face of emotional difficulties?
12	16	Have you ever felt that empathy prevents you from being objective in your evaluation?
13	17	How often do you discuss the emotional aspects of your work within your professional team?
14	19	Would you like to participate in empathy development or psychological support courses?
Research direction: the carryover of emotions from professional to personal life		
15	6	How would you describe the emotional impact of your work on your personal life?
16	12	Has your professional activity ever affected your dreams or sleep?
17	13	Do you feel that your perspective on your own health changes after being exposed to many cancer cases?
18	21	How do you feel about diagnosing cancer in young patients or children?
19	22	Would you recommend this profession to a young acquaintance, considering the emotional aspects, as well?

**Table 2:** Socio-demographic data of the study respondents.

Parameter	Respondents (*n*)	Respondents (%)
Age groups of respondents		
25–30 years old	29	17.58%
31–40 years old	58	35.15%
41–50 years old	35	21.21%
51–60 years old	39	23.64%
Over 60 years old	4	2.42%
Medical personnel category of respondents		
Laboratory technician	12	7.27%
Registry	2	1.21%
Resident Physician	36	21.82%
Specialist Physician	41	24.85%
Primary Physician	73	44.24%
Number of years of profession in pathology of the respondents		
Under 5 years	39	23.78%
5–10 years	38	23.17%
11–20 years	42	25.61%
21–30 years old	31	18.90%
Over 30 years	14	8.54%

**Table 3:** Answers provided by the respondents for the following research direction: Professional empathy in the absence of direct patient contact by percentage and numerical data.

Figure 1	Research Direction: Professional Empathy in the Absence of Direct Patient Contact	Answers (%)	Answers (*n*)	Total Answers
A	To what extent do you believe that the lack of direct contact with the patient affects your professional empathy?			
	It does not affect it	49.70%	82	165
	I don’t know/I haven’t thought about it	2.42%	4	
	It moderately affects it	41.21%	68	
	It deeply affects it	6.67%	11	
B	Do you consider the diagnostic process to be an empathic experience, even if the patient is unseen?			
	Yes, clearly	64.02%	105	164
	No	4.27%	7	
	Rarely	6.10%	10	
	Sometimes	25.61%	42	
C	Do you think of the patient as a person during histopathological processing (including viewing the slide under the microscope)?			
	Yes, always	52.73%	87	165
	Very rarely	7.88%	13	
	Sometimes, depending on the case	39.39%	65	
	Never	0.00%	0	
D	Have you ever felt the need to know more about the personal story of the patient whose pathology you are analyzing in the lab?			
	Yes, frequently	21.21%	35	165
	Very rarely	26.06%	43	
	Sometimes	46.06%	76	
	Never	6.67%	11	
E	Do you believe that medical training includes sufficient preparation for managing the emotional impact of the profession?			
	Not at all	36.36%	60	165
	Insufficient	37.58%	62	
	Partially	18.18%	30	
	Yes	7.88%	13	
F	How important is empathy in pathology practice, in your opinion?			
	Essential	25.45%	42	165
	Unimportant	1.82%	3	
	Important	54.55%	90	
	Slightly important	18.18%	30	

**Table 4:** Answers provided by the respondents for the following research direction: The emotional impact of oncologic diagnosis on medical personnel in pathology departments (1) by percentage and numerical data.

Figure 2	Research Direction: The Emotional Impact of Oncologic Diagnosis on Medical Personnel in Pathology Departments. (1)	Answers (%)	Answers (*n*)	Total Answers
A	Do you ever feel a form of emotional responsibility for the cancer diagnosis you establish?			
	Yes, frequently	63.64%	105	165
	Rarely	8.48%	14	
	Never	3.03%	5	
	Sometimes	24.85%	41	
B	Do you feel the need for dedicated psychological support for pathology staff?			
	Yes, it would be helpful	57.58%	95	165
	Yes, urgently	1.82%	3	
	No	17.58%	29	
	I’m not sure	23.03%	38	
C	How often are you emotionally affected by a complex or advanced cancer diagnosis?			
	Very often	22.42%	37	165
	Rarely	13.94%	23	
	Sometimes	61.82%	102	
	Never	1.82%	3	
D	Do you believe your work involves a component of moral distress?			
	Yes, constantly	23.17%	38	164
	No	10.98%	18	
	Rarely	14.02%	23	
	Sometimes	51.83%	85	

**Table 5:** Answers provided by the respondents for the following research direction: The emotional impact of oncologic diagnosis on medical personnel in pathology departments (2) by percentage and numerical data.

Figure 3	Research Direction: The Emotional Impact of Oncologic Diagnosis on Medical Personnel in Pathology Departments (2)	Answers (%)	Answers (*n*)	Total Answers
A	How do you evaluate collegial support in the face of emotional difficulties?			
	Satisfactory	53.33%	88	165
	Nonexistent	13.94%	23	
	Unsatisfactory	16.36%	27	
	Excellent	16.36%	27	
B	Have you ever felt that empathy prevents you from being objective in your evaluation?			
	Yes	4.88%	8	164
	Rarely	39.02%	64	
	Sometimes	17.68%	29	
	Never	38.41%	63	
C	How often do you discuss the emotional aspects of your work within your professional team?			
	Very rarely	36.36%	60	165
	Frequently	10.91%	18	
	Never	12.12%	20	
	Occasionally	40.61%	67	
D	Would you like to participate in empathy development or psychological support courses?			
	Yes, definitely	25.45%	42	165
	Probably yes	38.79%	64	
	No	9.09%	15	
	I’m not sure	26.67%	44	

**Table 6:** Answers provided by the respondents for the following research direction: The carryover of emotions from professional to personal life by percentage and numerical data.

Figure 4	Research Direction: The Carryover of Emotions from Professional to Personal Life	Answers (%)	Answers (*n*)	Total Answers
A	How would you describe the emotional impact of your work on your personal life?			
	Constant and difficult	18.79%	31	165
	Nonexistent	1.82%	3	
	Insignificant	10.91%	18	
	Variable, but manageable	68.48%	113	
B	Has your professional activity ever affected your dreams or sleep?			
	Yes, occasionally	40.61%	67	165
	Yes, frequently	11.52%	19	
	Rarely	32.12%	53	
	Never	15.76%	26	
C	Do you feel that your perspective on your own health changes after being exposed to many oncologic cases?			
	Yes, significantly	34.55%	57	165
	Sometimes	44.85%	74	
	Rarely	11.52%	19	
	No	9.09%	15	
D	How do you feel about diagnosing cancer in young patients or children?			
	Extremely difficult	40.61%	67	165
	I try not to think about it	3.64%	6	
	The same as in any case	5.45%	9	
	More difficult than in adults	50.30%	83	
E	Would you recommend this profession to a young acquaintance, considering the emotional aspects, as well?			
	Yes, with reservations	36.97%	61	165
	Yes, without reservations	49.09%	81	
	No	4.85%	8	
	I’m not sure	9.09%	15	

**Table 7:** Correlations between different variables in terms of sociodemographic characteristics and the survey questions.

No.	Order in the Questionnaire	Question Statement	Variable	*p*-Chi-Squared Test
1	4	To what extent do you believe that the lack of direct contact with the patient affects your professional empathy?	Personal category	0.0016
2	7	Do you consider the diagnostic process to be an empathic experience, even if the patient is unseen?	Resident versus specialist or consultant	0.0446
3	8	Do you think of the patient as a person during histopathological processing (including viewing the slide under the microscope)?	Professional experience	0.0267
			Personal category	0.000065
			Resident versus specialist or consultant	<0.0001
4	14	Have you ever felt the need to know more about the personal story of the patient whose pathology you are analyzing in the lab?	Resident versus specialist or consultant	0.0151
5	18	Do you believe that medical training includes sufficient preparation for managing the emotional impact of the profession?	Professional experience	0.0118
			Personal category	0.000007
			Resident versus specialist or consultant	0.0021
6	5	Do you ever feel a form of emotional responsibility for the cancer diagnosis you establish?	Personal category	0.0000049

The result was considered statistically significant if the *p*-value was less than 0.05 (*p* < 0.05).

## Data Availability

Data is available on request to the first author.
